# Understanding temperature effects on recruitment in the context of trophic mismatch

**DOI:** 10.1038/s41598-019-51296-5

**Published:** 2019-10-23

**Authors:** T. Régnier, F. M. Gibb, P. J. Wright

**Affiliations:** Marine Scotland Science, 375 Victoria Road, Aberdeen, AB11 9DB Scotland UK

**Keywords:** Marine biology, Projection and prediction, Phenology, Fisheries

## Abstract

Understanding how temperature affects the relative phenology of predators and prey is necessary to predict climate change impacts and recruitment variation. This study examines the role of temperature in the phenology of a key forage fish, the lesser sandeel (*Ammodytes marinus*, Raitt) and its copepod prey. Using time-series of temperature, fish larval and copepod abundance from a Scottish coastal monitoring site, the study quantifies how thermal relationships affect the match between hatching in sandeel and egg production of its copepod prey. While sandeel hatch time was found to be related to the rate of seasonal temperature decline during the autumn and winter through effects on gonad and egg development, variation in copepod timing mostly responded to February temperature. These two temperature relationships defined the degree of trophic mismatch which in turn explained variation in local sandeel recruitment. Projected warming scenarios indicated an increasing probability of phenological decoupling and concomitant decline in sandeel recruitment. This study sheds light on the mechanisms by which future warming could increase the trophic mismatch between predator and prey, and demonstrates the need to identify the temperature-sensitive stages in predator-prey phenology for predicting future responses to climate change.

## Introduction

Predictions about the effect of climate change on marine ecosystems are limited by our understanding of processes affecting species and communities^1–3^. As most aquatic organisms are ectothermic, temperature has a direct effect on physiological rates such as metabolic^4,5^, growth^6^ and maturation^7^, leading to variation in mortality^8–10^, distribution^3,11,12^ and phenology^13^. While most studies have focused on direct temperature effects at the individual species level, diverging phenology among trophic levels may have a more profound impact at both the population and ecosystem level^14–16^. The majority of studies that have considered changing phenology have either inferred changes from the temporal variability in the occurrence of a single taxa such as fish larvae or copepods^17–19^ or compared general trends among trophic guilds across large spatial scales^20,21^. Neither of these approaches is amenable to identifying the mechanisms that lead to trophic mismatch, as information relating to variability in both the predator and prey responses is required. Consequently, it is important to understand how temperature affects the development of predator and prey and the impact this has on their respective phenology.

The importance of synchrony in predator-prey phenology is central to several hypotheses relating to the cause of variation in fish recruitment. Hjort^22,23^ identified a “critical period” as starvation at the transition between yolk and exogenous feeding is a key determinant of larval survival and a driver of year-class strength. Later, Cushing^24,25^ proposed that the synchrony or match between hatching and an appropriate phase of the prey production cycle was an important selective influence on early growth and mortality (the ‘match–mismatch’ hypothesis). These hypotheses appear most relevant to species that spawn near the onset of the spring bloom, when small changes in the timing of zooplankton production and fish hatching can lead to substantial changes in the available prey resource^26,27^. Direct empirical evidence for mismatch is limited however, especially in relation to putative climate effects. Low recruitment has been related to warmer winters in many temperate and boreal species^28–30^, although temperature can sometimes be a poor proxy for the prey resource available to winter hatching fish larvae^31^. Declines in the large calanoid copepod, *Calanus finmarchicus*, has been linked to low recruitment in North Sea cod^32^. However, Capuzzo *et al*.^20^ found a relationship between recent declines in recruitment of several North Sea fish species, including cod, and a decline in small calanoid copepod species. While these simple statistical relationships suggest an important climate influence, it is difficult to evaluate the biological significance as they do not shed light on the underpinning mechanisms. In contrast, the data requirements of models that account for all the biophysical and food web interactions needed to adequately represent climate-driven effects on larval survival can be too demanding and complex to provide robust estimates of recruitment. Hence, statistical relationships based on key processes can be worth exploring.

Due to their role as a key trophic link between secondary production and higher trophic levels, forage fish are an important determinant of marine ecosystem structure and stability, and climate-driven variations in their abundance have the potential to cascade-up and impact higher trophic levels^21,33^. In the North Sea, the lesser sandeel (*Ammodytes marinus*, Raitt) is an important forage fish that also supports the largest single-species fishery in the North Sea^34–36^. This species is a capital breeder that spawns in mid-winter with the larvae hatching near to the onset of the spring bloom^27,37^. Correlative studies have linked variation in sandeel recruitment to sea surface temperature, the North Atlantic Oscillation^30,38,39^, sea circulation pattern^39,40^ and density dependent effects through cannibalism from the previous year class^41,42^. However, bottom-up effects through prey availability appear likely based on local studies of plankton and early survival^27,43^ as well as some correlative and modelling studies^30,42,44^.

Recruitment appears to be established at a very early stage during the sandeel’s life cycle^43,45^, with the likely mechanism being the synchrony of first feeding larvae and the seasonal appearance of suitable sized prey^43^. Copepods of the *Calanus* genus have repeatedly been identified as an important prey for sandeel larvae and juveniles^46–48^ and the abundance of these have been found to be significant in regression models of recruitment^42,43^. Empirical evidence shows gape-size limitation in first feeding sandeel, and suggests selectivity towards the egg and early naupliar stages of the copepod^48,49^. In the northwest North Sea (ICES sandeel area SA4), sandeel recruitment variation could be explained by the degree of mismatch between *Calanus helgolandicus* egg production and sandeel hatch date^43^. Whether this relationship indicates a strict link between the two species, or whether *C*. *helgolandicus* phenology covaries with that of a range of copepod prey species, still needs to be determined.

Direct effects of temperature on sandeel physiological rates have been observed, with a negative relationship with oocyte maturation^50^ and a positive relationship with egg development^51^. The negative temperature effect on oocyte development is linked to an adult’s need to regulate energy usage throughout the autumn, both to survive buried in sand and to reproduce^52^.Their copepod prey also exhibits a positive temperature-dependent developmental rate^53^, leading to changes in phenology^14^. Therefore, as predator and prey responses to temperature differ, one can expect indirect temperature effects on sandeel recruitment mediated by a mismatch between the timing of sandeel hatching and that of the peak availability of their prey.

The North Sea is a rapidly changing environment, with an observed temperature increase of 0.6 to 1.5 °C in the last 3 decades^54^ having led to changes in the copepod community^55,56^. With a further projected increase of 0.2 to 0.3 °C per decade^57^, understanding how climate change affects the mechanisms responsible for variations in the recruitment of key trophic links is necessary to anticipate changes in the structure of North Sea communities.

Using a time-series of temperature, sandeel larval and copepod abundance, this study examined (i) the direct role of temperature on the phenology of both predator and prey, (ii) the resulting temporal match between sandeel hatching and egg production in *C*. *helgolandicus* and other abundant calanoid copepods, (iii) the ability of temperature change to predict trophic mismatch and recruitment in sandeel and (iv) the implication of climate projections on the future synchrony in the seasonal appearance of sandeel and their prey. Finally, we discuss how regression based approaches targeted on key life-stages of predator and prey phenology can enable prediction of mismatch by accounting for the underlying mechanisms.

## Results

### Predator-prey phenology

**Figure 1:**
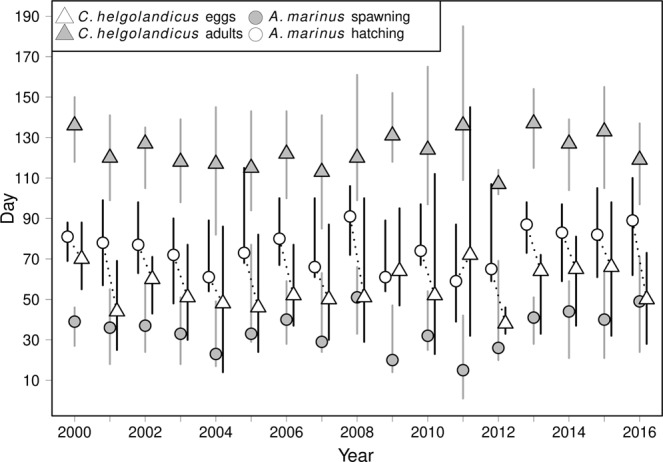
Inter-annual variations in *C*. *helgolandicus* and sandeel phenology. The mismatch measure is indicated by the dotted line between the median day of *C*. *helgolandicus* egg production and the median day of sandeel hatching. The bars correspond to 95% CIs.

The dates of peak spring abundance of the adult stage of *C*. *helgolandicus* were significantly correlated with the dates of peak spring abundance of *Pseudocalanus sp* (r = 0.58, p = 0.01) and *Acartia clausi* (r = 0.57, p = 0.02) but not *Paracalanus parvus* (r = 0.41, p = 0.1) and *Temora longicornis* (r = 0.32, p = 0.2). This result indicates that a number of copepod species show a similar pattern of annual variations in their seasonal peak. Spring peak in abundance of reproductive stages (cVI stage) of *C*. *helgolandicus* showed inter-annual variation, as the date of maximum abundance varied between calendar day 107 and 137 (starting between day 82 and 118, and finishing between day 114 and 185; Fig. 1). Back-calculated dates of egg production were also variable, with a maximum between day 38 and 72, while starting as early as day 14 and finishing as late as day 145; Fig. 1). These predictions are in agreement with the recorded presence of large naupliar stages in the zooplankton time series in the period corresponding to the back-calculated period of naupliar development in *C*. *helgolandicus* (Supplementary Fig. S1). Based on the measured abundance of larvae, hatching in sandeel was also variable and took place between day 39 and 115 while the date at 50% hatching varied between day 59 and 91 (Fig. 1). Accordingly, back-calculated spawning dates were centred between day 15 and day 51 and lasted from the first day of the year to day 77 (Fig. 1).

### Direct temperature effects on phenology

**Figure 2:**
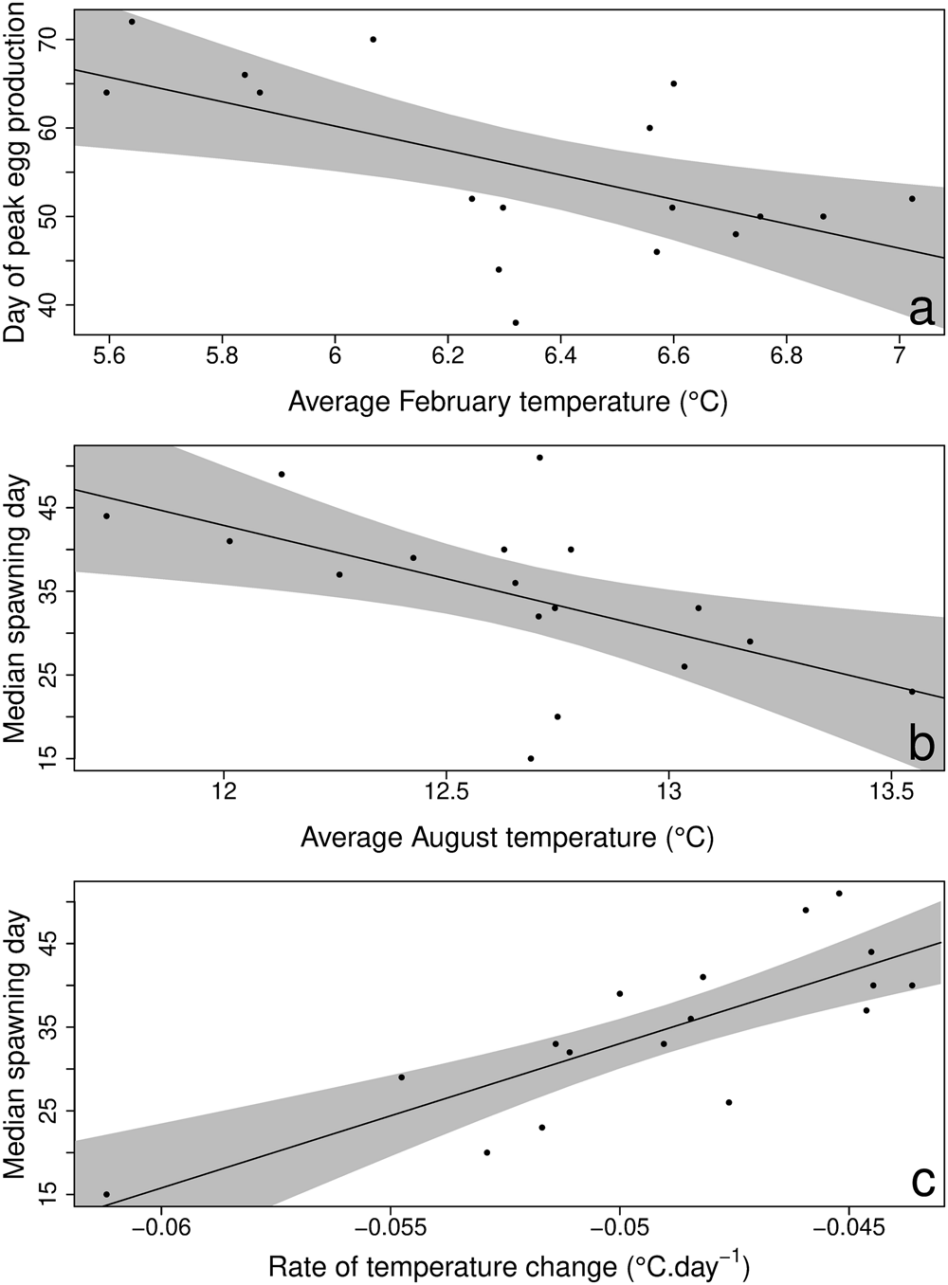
Relationships between predator-prey phenologies and environmental variables. (**a**) Influence of average February temperature on *C*. *helgolandicus* peak egg production. Effect of (**b**) average August temperature and (**c**) the rate of temperature decrease (September to February) on sandeel median spawning date.

Median dates of *C*. *helgolandicus* egg production were significantly related to monthly average temperatures for the winter months of December, January and February (Table 1). Peak egg production date was negatively related to sea water temperature during these months (Fig. 2a). In contrast, median dates of sandeel spawning were only significantly related to average temperatures during the preceding month of August (Table 1) with earlier spawning associated with high August temperatures (Fig. 2b). Median spawning was also influenced by the temperature difference between September and February but most significantly, by the linear rate of temperature decrease between September and February (Table 1, Fig. 2c). Due to a large part of spawning date variation being explained by the linear rate of temperature decrease, the effect of average August temperature disappeared when both were introduced in the model.

**Table 1:** Linear regressions between monthly average sea temperatures and key phenological events in *Calanus helgolandicus* and sandeel.

DV	Month	F	df.num	df.den	R^2^	*p-value*
Peak in *C*. *helgolandicus* egg production	May (year-1)	2.77	1	15	0.16	0.12
	June(year-1)	0.35	1	15	0.02	0.56
	July(year-1)	2.63	1	15	0.15	0.13
	August(year-1)	4.24	1	15	0.22	0.06
	September(year-1)	0.98	1	15	0.06	0.34
	October(year-1)	1.13	1	15	0.07	0.30
	November(year-1)	3.14	1	15	0.17	0.10
	***December(year-1)***	***7.59***	***1***	***15***	***0.34***	***0.01***
	***January***	***6.65***	***1***	***15***	***0.31***	***0.02***
	***February***	***8.18***	***1***	***15***	***0.35***	***0.01***
	March	0.10	1	15	0.01	0.76
Sandeel spawning date	May (year-1)	0.33	1	15	0.02	0.58
	June(year-1)	1.01	1	15	0.06	0.33
	July(year-1)	3.34	1	15	0.18	0.09
	***August(year-1)***	***7***.***54***	***1***	***15***	***0***.***33***	***0***.***02***
	September(year-1)	4.52	1	15	0.23	0.05
	October(year-1)	0.33	1	15	0.02	0.57
	November(year-1)	0.12	1	15	0.01	0.74
	December(year-1)	2.93	1	15	0.16	0.11
	January	2.37	1	15	0.14	0.14
	February	1.76	1	15	0.10	0.20
	March	0.10	1	15	0.01	0.76
	Average temperature (Sept-Feb)	0.61	1	15	0.04	0.45
	***Temperature Difference (Sept-Feb)***	***9***.***9***	***1***	***15***	***0***.***36***	***0***.***007***
	***Linear rate of Decrease (Sept-Feb)***	***26***.***64***	***1***.***00***	***15***.***00***	***0***.***64***	<***0***.***001***

### Indirect temperature effects on mismatch

**Figure 3:**
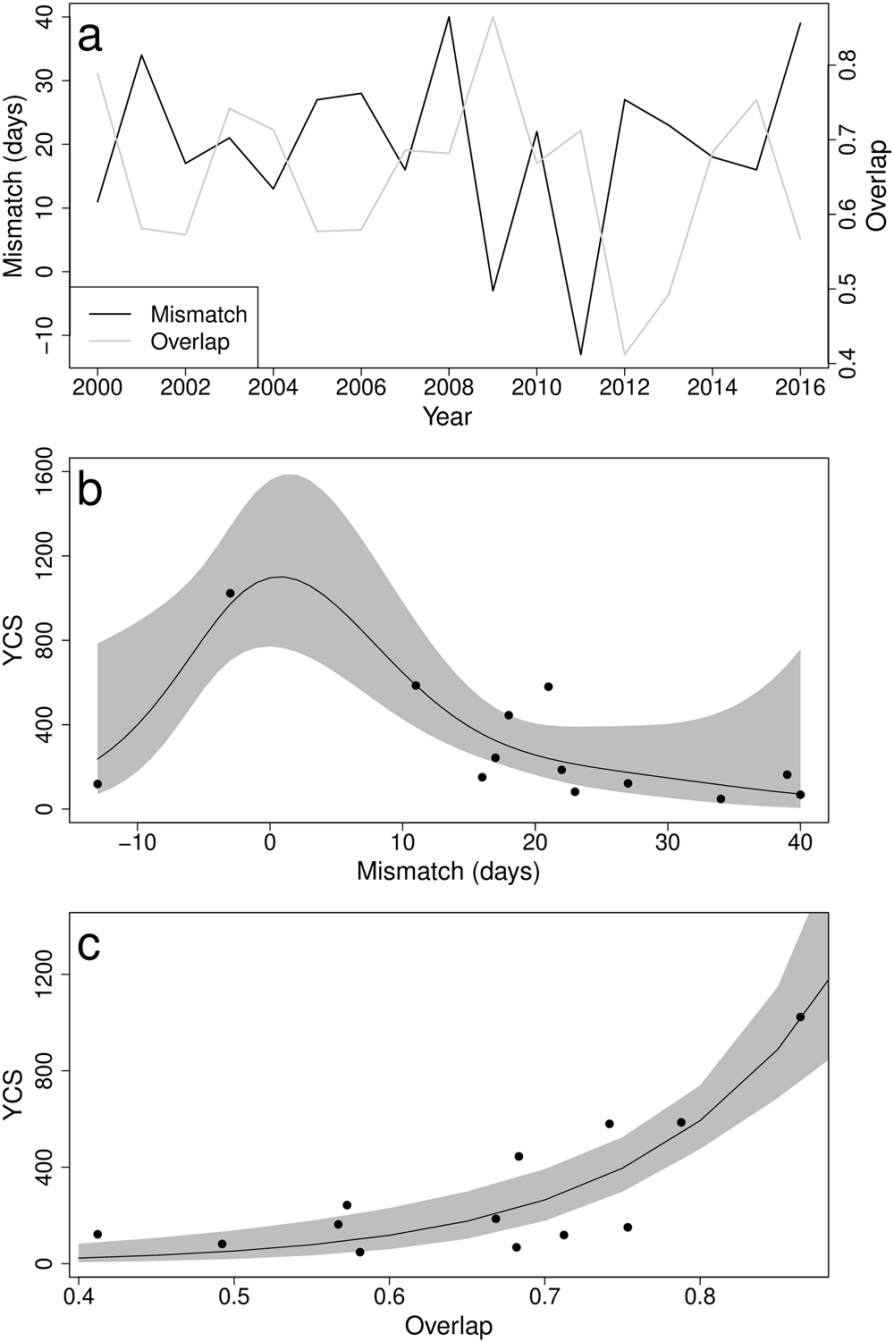
Variations in the mismatch and overlap measures. (**a**) Inter-annual variations and relationships between sandeel recruitment index (YCS) and (**b**) the mismatch measure and (**c**) the overlap measure. 95% CIs are indicated by the grey area around the mean prediction (solid line).

**Figure 4:**
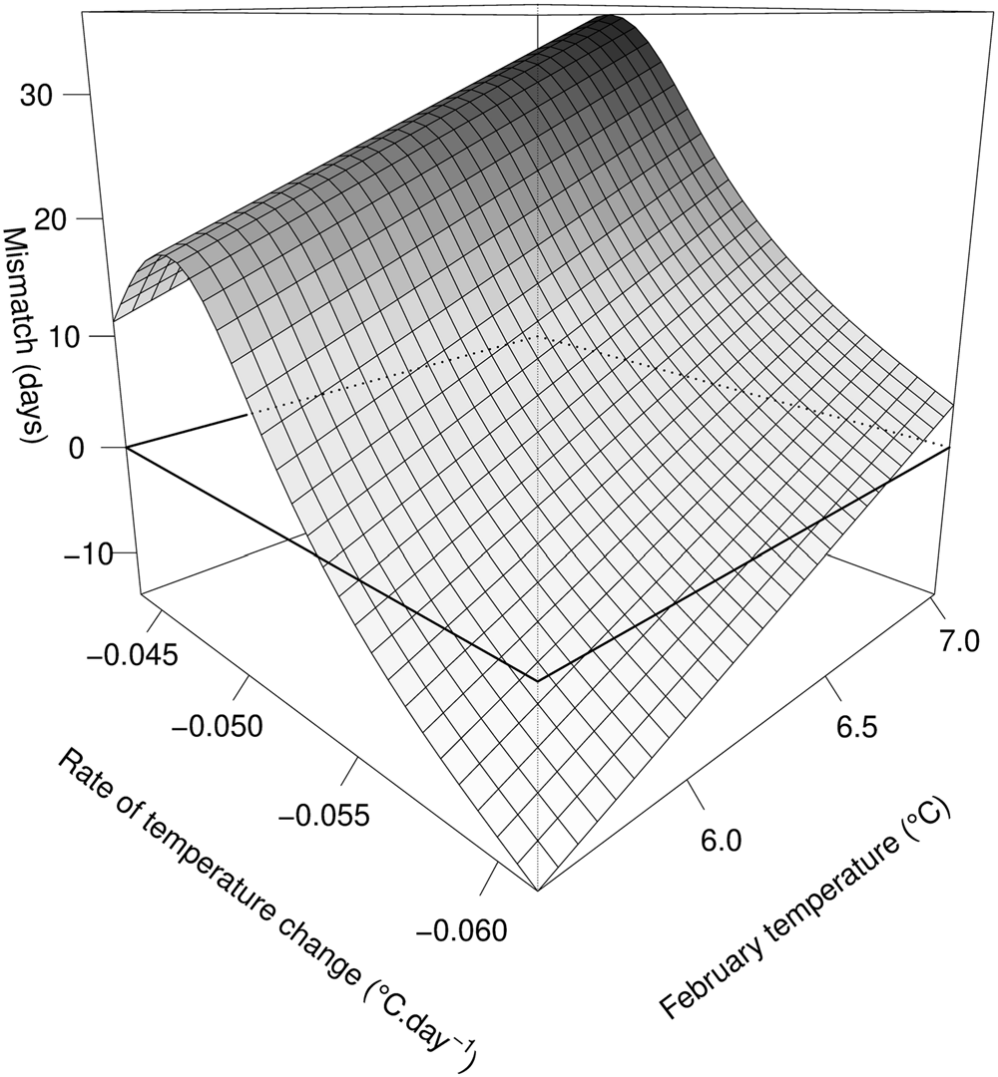
Relationship between the mismatch measure, the rate of temperature decrease (September-February) and average February temperatures. Perfect synchrony between predator and prey is indicated by the intersecting plan corresponding to a 0 mismatch.

Both the mismatch and the overlap measures showed substantial inter-annual variation (Fig. 3a). Indeed, the mismatch measure indicated that in 2011, the central date of sandeel hatching preceded *C*. *helgolandicus* egg production by 13 days while in 2008, *C*. *helgolandicus* egg production preceded sandeel hatching by 40 days. The overlap between sandeel hatching and the egg production of their copepod prey was minimal in 2012 with a value of 0.41, and maximum value in 2009 of 0.86 (Fig. 3a). Overall, the overlap was maximum when the mismatch measure was close to 0 (Fig. 3), indicating synchrony in both predator and prey phenologies. The mismatch measure was related to the rate of temperature decrease between September and February, using a smooth term, and linearly to average February temperature (Table 2, Fig. 4). The overlap, however, was neither solely influenced by the rate of temperature decrease between September and February or average February temperature (Table 2).

**Table 2:** Model outputs of the dependence of the mismatch measure, the overlap and the recruitment index (year class strength) on environmental and stock variables.

1-GAM- Mismatch	Explained Deviance: 74.4%			
**Parametric coefficients**	**Estimate**	**Std.Error**	**t-value**	**Pr(>|t|)**
Intercept	−59.753	33.198	−1.8	0.09
Average February temperature	12.536	5.225	2.4	0.03*
**Smooth terms**	**Edf**	**Ref.df**	**F**	***p*** **-value**
s(Rate of temperature decrease)	2.804	3.272	5.798	0.008**
**2-GLM- Overlap**	**Residual Deviance = 0.82 on 14d.f (pseudo R** ^**2**^ **= 0.11)**			
**Parametric coefficients**	**Estimate**	**Std.Error**	**t-value**	**Pr(>|t|)**
Intercept	0.162	2.856	0.057	0.95
Average February temperature	−0.161	0.317	−0.506	0.62
Rate of temperature decrease	−30.289	30.369	−0.997	0.34
**3-GAM- Sandeel recruitment (YCS)**	**Explained Deviance: 91%**			
**Parametric coefficients**	**Estimate**	**Std.Error**	**t-value**	**Pr(>|t|)**
Intercept	5.357	0.2271	23.6	<0.001***
Age1 abundance	−5.8 × 10^−5^	1.22 × 10^−4^	−0.476	0.65
**Smooth terms**	**Edf**	**Ref.df**	**F**	***p*** **-value**
s(mismatch)	3.363	3.761	14.4	0.001**
**4-GLM- Sandeel recruitment (YCS)**	**Residual Deviance = 254756 on 11d.f (pseudo R** ^**2**^ **= 0.72)**			
**Parametric coefficients**	**Estimate**	**Std.Error**	**t-value**	**Pr(>|t|)**
Intercept	−0.08	1.26	−0.064	0.95
Overlap	8.08	1.56	5.18	0.0003***

The index of sandeel recruitment was related to both measures of mismatch and overlap but not to the abundance of age 1 sandeel (Table 2). A non-linear relationship, analysed with a GAM (Table 2), best described the effect of the mismatch measure on sandeel recruitment, with a local maximum in recruitment indices corresponding to synchrony in predator and prey phenologies (i.e. when mismatch is 0, Fig. 3b). The relationship between sandeel recruitment and the overlap was best described using a GLM (Table 2) due to the monotonous increase in recruitment index with the overlap measure (Fig. 3c).

### Mismatch and future climate

**Figure 5:**
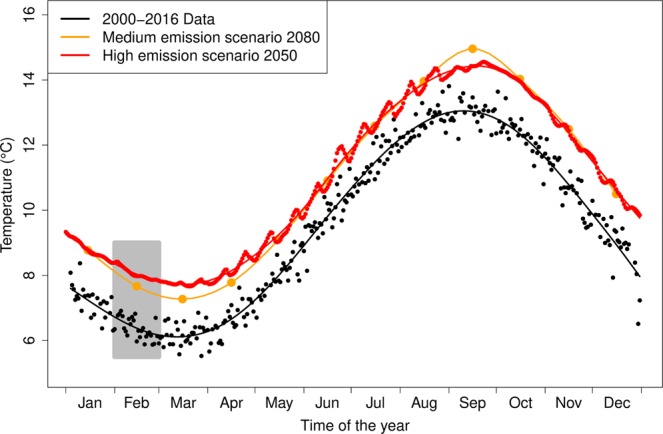
Seasonal variations in seawater temperature near the seabed at the Stonehaven monitoring station, in the recent past and according to two climate change scenarios. Observed temperatures between 2000 and 2016 are indicated in black, projections using a medium green house gas emissions scenario in orange and high green house gas emissions scenario in red. February temperatures are indicated by a grey box.

The climate projections used predicted an increase of 1.3 °C in average February temperatures for the medium greenhouse gas (GHG) emission scenario, and 1.7 °C for the high GHG emission scenario (Fig. 5). The linear rate of temperature decrease between September and February were within the range observed in present years. As a result, while the phenology of sandeel is predicted to remain similar to the present observations, *C*. *helgolandicus* egg production is predicted to occur between 18 and 24 days earlier than the present average (Table 3). As a consequence, the mismatch between sandeel and their prey is predicted to increase by 10 to 22 days with medium and high GHG scenarios respectively (Table 3) and result in poor sandeel recruitment.

**Table 3:** Comparisons of peak egg production in *C*. *helgolandicus*, spawning day and embryonic development in sandeel and the resulting trophic mismatch in present temperature conditions or predicted according to modelled future climate projections. Numbers in brackets correspond to 95% CIs.

Climate change scenario	*C*. *helgolandicus* peak egg production	Sandeel spawning	Sandeel average incubation duration	Mismatch
*Present data (2000*–*2016)*	55.5 (40.4–71.2)	34.6 (17–50.2)	40.6 (37.8–44.4)	19.8 (−9–39.6)
*Average GHG emissions 2080*	37.3 (24.2–50.3)	28.6 (24.9–32.2)	34.4 (33.1–34.8)	29.3 (14.5–44.1)
*High GHG emissions 2050*	31.7 (15–48.5)	31.9 (28.9–35)	32.1 (32.1–33)	41.4 (22.2–60.5)

## Discussion

The present study was able to predict the degree of trophic mismatch from temperature alone based on an understanding of temperature dependency of sandeel and copepod development. While the importance of temperature on phenology in marine communities is widely recognised^14,58^, few empirical studies that have invoked mismatch have considered the temperature dependent processes affecting predator and prey development rates. The need for a precise understanding of the nature and timing of processes is well illustrated from studies of North Sea cod, as although many temperature and copepod relationships have been proposed^32,59^, recent modelling indicates that recruitment is only influenced by larval growth rate around the peak hatch^60^. So, as the present study highlights, it is important to account for variability in the time-specific influences on both larval hatching times and zooplankton egg production.

The relative peak timing of fish larvae and copepod eggs (mismatch measure) was found to be significantly influenced by a combination of the linear rate of temperature decrease between September and February and average February temperatures. It is important to note that as no plankton sampling method is free of sampling bias due to both spatial and temporal variations in abundance at the sampling site, this bias might affect the back-calculated phenology. However, the recorded seasonal pattern of abundance was consistent with records obtained at other coastal monitoring sites^61^. These temperature effects on mismatch correspond to the direct effects on the phenologies of sandeel and *C*. *helgolandicus* respectively. Variation in the timing of spawning could be explained by the linear rate of temperature decrease between September and February. During this period, sandeels leave the water column^62,63^ and bury in the sand until winter spawning^64,65^. The start of this period, in August - early September^63^, coincides with the phase of exogenous vitellogenesis marking a change in energy allocation^66^. As energy is allocated to gonad maturation and diverted from somatic maintenance during their buried phase, sandeels continually have to adjust their reproductive allocation in response to metabolic demands^52^. Accordingly, above average temperatures are associated with later spawning^50^ and so the rate of cooling from the early autumn peak should reflect the rate of gonad development.

Increasing temperature may lead to more favourable growth conditions^56^ and earlier egg production as development time and egg production rate are temperature dependent in *C*. *helgolandicus*^53^. In particular, temperatures above 6 °C may be essential for growth in *C*. *helgolandicus*^56^, which in the study area was not always exceeded and could have led to delays in egg production. Variation in *C*. *helgolandicus* spring phenology was significantly correlated to the timing of peak adult abundance of two smaller sized copepods: *Pseudocalanus sp* and *Acartia clausi*. These species are representative of the southern North Sea copepod community^20,67^, have similar temperature dependent generation times to *C*. *helgolandicus*^53,68,69^, and their nauplii have been observed in February and March in the study area^70^. Similar trends in species abundance have been reported in a previous North Sea study^33^. Thus the apparent dependence of sandeel recruitment on the synchrony between hatching and *C*. *helgolandicus* egg production (e.g.^43^, the present study) may well be associated more with a wider community of copepods showing a similar trend in their spring phenology.

Consistent with^43^, the mismatch between the timing of sandeel hatching and back-calculated peak in egg production of the calanoid copepod, *C*. *helgolandicus*, was a good predictor of variation in sandeel recruitment. While the relationship found previously was based on 7 observations^43^, the present study confirms the robustness of this association with an improved time-series composed of 13 years. Due to the small sample size and absence of negative mismatch values, the relationship found in the previous study^43^ was best described by a linear relationship. The GAM model used in the present study reveals that high recruitment is dependent on a close match in the phenology of predator and prey, and conversely that recruitment is low for both positive and negative mismatch, in line with theoretical expectations^71,72^. In addition, a second measure of mismatch, the overlap index, based on the coincidence between the distribution of sandeel hatching and the distribution *C*. *helgolandicus* egg production, confirmed the importance of synchronous phenology on sandeel recruitment. Recruitment variation in this study was not considered relative to spawning stock biomass, as no significant relationship has been found^43,73^. Further, there was no evidence for the type of negative density dependent effect found in the central and southern North Sea sandeel stock (SA1)^30,42^. Rather, periods of low and successively above average recruitment in the SA4 stock point to strong environmental forcing, as has also been seen in the Shetland stock^74^.

The two temperature effects on the mismatch measure allowed for predictions of mismatch under different climate change scenarios. While the average mismatch in the recent past (2000–2016) is 19.8 days, a medium GHG emission scenario would result in a 48% increase in mismatch by 2080, while a high GHG emission scenario would lead to a 110% increase even sooner, by 2050. Thus, if the temperature-dependence of the mismatch measure extends outside of the temperature range considered in this study, *C*. *helgolandicus* (or other copepods showing a similar temperature response), would cease to be available as a prey resource for first feeding sandeel larvae. While these results should be considered with caution as the predicted temperature range is outside the observed range on which these relationships were quantified, one can speculate on a number of non-mutually exclusive possible outcomes. In a worst case scenario, sandeel larvae might fail to meet their energy requirements at first feeding, and suffer high mortality rates, which might ultimately lead to their disappearance from this region of the North Sea. Due to modified interactions between species, regime shifts may occur^75^, where changes in bottom-up and top down processes may result in a rapid transition of the North Sea ecosystem to a different state. Such regime shifts are likely to happen when species undergo climate-driven range shifts, as the expansion/contraction of species range leads to new species interaction. For instance, the replacement of the boreal *Calanus finmarchicus* by the temperate *C*. *helgolandicus* in some regions of the North Sea resulted from a climate driven northward expansion of the latter^76^. Similar shifts have been observed in forage fish^11^ with warmer temperatures and a resulting extended feeding season may favour income breeding species such as sprat or anchovies^77^ over sandeel. While genetic adaptation is unlikely on such a short timescale, an adaptive response of sandeel to climate change is possible, as trans-generational plasticity (e.g. maternal effects) may allow the resilience of sandeel populations and provide time for genetic adaptation to take place^78,79^, In particular, variation in female gonad maturation has been linked to phenotype^64^ and may provide a buffer against variations in the timing of food availability for offspring.

There have been many attempts to explain sandeel recruitment and the subsequent effect it has on sandeel reliant seabirds using simple regression approaches incorporating temperature and adult *Calanus* abundance^42,80,81^, but these have proved to be unreliable on further inspection^31,82^. Key to the significance of the relationship reported here is that it takes account of known temperature dependent development relationships. However, while the effect of sandeel and *C*. *helgolandicus* synchrony on sandeel recruitment was also observed in a comparatively extended time series^43^ with naupliar stages of large copepods being recorded at times consistent with the back-calculated phenologies, further work is needed to confirm the key prey assemblage through stomach content analyses of sandeel larvae and analysis of the winter micro-zooplankton community. Although temperature often explains a high proportion of inter-annual variation in copepod timing^19^, the importance of primary production on copepod egg production should also be taken into account, probably as a threshold response as indicated from field relationships^56,70^.

The substantial variation in synchrony between sandeel larvae and their copepod prey found in the present study indicates a greater potential for mismatch than that suggested by larger scale overviews of the annual variation in copepod and fish larval timing^14,19^. The estimated peak and range in hatch dates based on larval occurrence were consistent with that seen in other parts of the North Sea^18,45,83^ and to back-calculations from otolith daily increment counts^27,43^. Back-calculated spawning dates were also consistent with direct observations^64,65,84^. The estimated timing of *C*. *helgolandicus* egg production is less certain but females with eggs have been observed at these dates and temperatures^61,85–88^, and *Pseudocalanus* nauplii have been recorded at this time^70^. Although this study focusses on just one of a number of possible climate change stressors, it does demonstrate that regression based approaches can provide indicators of climate change on a key trophic relationship. The approach adopted in this study could be adapted to other sandeel stocks and winter spawning species. However, in contrast to sandeels, reproductive development rate in many fish that spawn in winter is positively related to temperature^89^. Consequently, the direction of phenological change in spawning time^13,90^ is generally the same as that of zooplankton production. Evidence suggests that the magnitude of changes in phenology tends to be larger in copepods than fish^19^ and further down the trophic chain, primary producers and consumers display even faster rates of phenological responses to climate^91^. Future climate projections suggest a further decrease in the synchrony of predator-prey life cycles. Therefore, an ecosystem approach to management requires the effects of climate and exploitation to be accounted for not only on the target species, but also on related trophic levels.

## Methods

### Environmental and biological data

**Figure 6:**
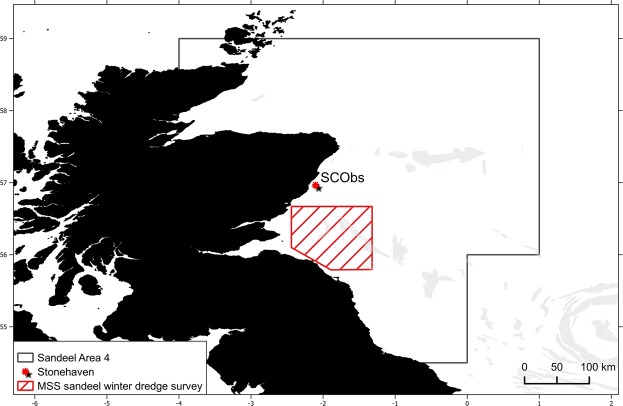
Study Area. Boundary of sandeel management area 4 is indicated by a solid black line, the location of MSS sandeel winter dredge surveys by red shading and the Scottish Coastal Observatory (SCObs) station of Stonehaven by a red star.

This study focuses on the southern part of sandeel management area 4 (Fig. 6) in which time-series of physical and biological data are collected at a monitoring site and where surveys dedicated to the assessment of sandeel abundances in the management area take place. The area covered by this study is inferior to the spatial extent of sandeel population processes in the region^92,93^ and therefore relevant to infer the role of environmental drivers on recruitment. Sea temperature data, abundances of 5 copepod species and abundances of newly hatched sandeel larvae were collected weekly between 2000 and 2016 at the MSS coastal ecosystem monitoring site on the Scottish East coast at Stonehaven (http://data.marine.gov.scot/dataset/scottish-coastal-observatory-stonehaven-site, Fig. 6). Samples to measure temperature were collected using a reversing bottle and digital thermometer on the sea bed (~45 m) and near the surface. As tidal stirring mixes the water column and the water column is not stratified at the sampling site^94^, bottom temperatures are used in subsequent analyses. Weekly samples of various zooplankton taxa were collected using 40 cm diameter bongo nets fitted with 200 μm mesh^94^. The nets were hauled vertically from near bottom to surface at a speed of 2–3 m.s^−1^ and as such, sampled the zooplankton across the entire water column. Sandeel larvae and juveniles appear to preferentially feed on naupliar and copepodite stages of the *Calanus* genus^49,95^ and a previous study identified *Calanus helgolandicus* as an important resource for larval survival and recruitment^43^, thus the spring phenology of this large copepod was estimated in comparison to sandeel phenology. However, as smaller copepod species are considered more representative of the North Sea copepod community^20,96^, the timing of adult peak abundance for *Paracalanus parvus*, *Pseudocalanus sp*., *Temora longicornis* and *Acartia clausi* was estimated and compared to the phenology of *C*. *helgolandicus* to assess if temperature-induced changes in phenology are similar at zooplankton community level or vary between taxa. Adult stage abundances (copepodite VI stage) were used as only this stage allows morphological identification at the species level.

Weekly sampling of sandeel larvae took place using a 1 m ring net, fitted with a 350 μm mesh net, towed obliquely from a depth within 3 m of the seabed (~45 m) to the surface. Sandeel larvae were measured and abundance <8 mm total length (corresponding to fish that have hatched within 10 days of capture date; Régnier *et al*., 2018) used to determine hatch date distributions for each of the 17 years under study. Abundances of both newly hatched larvae and the 5 copepod species were used to define the start (5% cumulative distribution), peak (50% cumulative distribution) and end (95% cumulative distribution) of these key events of the phenology of both predator and prey. Abundances of age 0 and age 1 sandeel were derived from the annual MSS sandeel winter dredge survey^97^, off the Firth of Forth area (Fig. 6), for the available corresponding years (2000–2003 and 2008–2016). The abundance index of age 0 fish was used as a measure of recruitment (year class strength), and abundance of age 1 sandeel was used to test for density dependent effects on recruitment, as previously reported^38,42^.

### Predator/prey phenology

The timing of *C*. *helgolandicus* egg production and sandeel spawning were back-calculated using sea temperature data collected weekly in Stonehaven, smoothed abundances of *C*. *helgolandicus* cVI stages and sandeel larvae, and published relationships between development duration and temperature in these two species. Weekly sea temperature measures between 6^th^ January 1999 and 19^th^ December 2016 were modelled using a Generalized Additive Model (GAM) with a thin plate smoothing term on the date and a high basis dimension (90), as it provided an excellent fit (R^2^ = 0.98) to the temperature data and allowed sensible extrapolation at the daily level (T_d_).

The timing of *C*. *helgolandicus* egg production (EP.Ch) was estimated as:

1 $$EP.\,C{h}_{d-D{T}_{Ch}}={{\rm{cVI}}}_{d}$$

where cVI_d_ is copepodite VI stage abundance on day d, and:

2 $$D{T}_{Ch}=\frac{1}{{{\rm{b}}}_{{\rm{i}}}{({\rm{T}}+{{\rm{T}}}_{0})}^{2.05}}$$

where DT_Ch_ is development time between the egg and cVI stage, T is temperature, T_0_ a constant, specific to *C*. *helgolandicus* (6.01^53^, b_i_ is a stage-specific coefficient (8.106 × 10^−5^ ^53^; and the 2.05 exponent characterises the temperature response of a large range of copepod species^98^. However, as temperature fluctuated during the course of *C*. *helgolandicus* development, the estimated daily temperature (T_d_) was used and equation [2] published by Cook *et al*.^53^ was expressed as developmental rate (DR_Ch_) in percent per day, such that:

3 $$D{R}_{Ch}=100\times {b}_{i}{({T}_{d}+{T}_{0})}^{2.05}$$

and for *C. helgolandicus* hatched on day *d*, DT_Ch_ is solved when ${\sum }_{d}^{d-D{T}_{Ch}}D{R}_{Ch}=100 \%$.

Calculations were based on copepodite cVI abundances as this stage is the most reliable for species identification in copepods. As consecutive generations may accumulate at this stage and therefore bias the back-calculation of egg production due to the presence of older generations, the observed dates of peak abundance in the earlier cV stage were compared to the back-calculated dates of cV peak abundances from observed cVI abundances. The results (Supplementary Table S1 and Supplementary Fig. S2) indicate a good match between observations and predictions with differences inferior to the sampling intervals on average.

Similarly, for sandeel, egg production (EP.Am) was back-calculated from hatch date distributions as:

4 $$EP.\,A{m}_{d-I{D}_{Am}}={{\rm{L}}}_{d.Am}$$

where *L*_*d*.*Am*_ is newly hatched sandeel larvae abundance on day *d* and *ID*_*Am*_ is incubation duration, solved when ${\sum }_{d}^{d-I{D}_{Am}}D{R}_{Am}=100 \%$.

With DR_Am_ the temperature dependant developmental rate of sandeel during incubation (in percent per day) was obtained from^51^ and expressed as:

5 $$D{R}_{Am}=0.51\times {{T}_{d}}^{0.85}$$

### Mismatch measures

In subsequent analyses of the influence of environmental variables on *C*. *helgolandicus* and sandeel phenologies, the median date of egg production was considered. Similarly, for each year *y*, a measure of trophic mismatch (in days) between sandeel and *C*. *helgolandicus* hatching was calculated as:

6 $$Mismatc{h}_{y}=med\,{L}_{d.A{m}_{y}}-med\,EP.C{h}_{y}$$

where, *med Ld*.*Am*_*y*_ and *med EP*.*Ch*_*y*_ are the median dates of hatching and egg production in year *y* for sandeel (*A*. *marinus*) and *C*. *helgolandicus* respectively.

Another index of mismatch representing the overlap between the distributions of sandeel and *C*. *helgolandicus* hatching dates, was calculated as:

7 $$Overla{p}_{y}=\alpha .\frac{Am.duratio{n}_{y}+Ch.duratio{n}_{y}}{Total\,duratio{n}_{y}}$$

where *Am*.*duration*_*y*_ and *Ch*.*duration*_*y*_ are the durations in days of the hatching periods of sandeel and egg production of *C*. *helgolandicus* respectively, calculated as the number of days between the date at which 5% and 95% of the respective cumulative distributions each year *y*. *Total duration*_*y*_ corresponds to the duration of the hatching period of sandeel and *C*. *helgolandicus* combined (number of days between the date of 5% hatching for whichever species hatched first and the date of 95% hatching for whichever species hatched last). A standardising coefficient α equal to 1/2 was used so overlap varied between 0 (no overlap) and 1 (perfect overlap).

### Statistical analyses

The temperature variables for the 17 years investigated comprised average monthly temperatures. To account for temperature changes during the sandeel maturation period^66^, the average temperature and the temperature difference between September and February as well as the linear rate of temperature decrease between September and February were used. The linear rate of temperature decrease was calculated with the *mblm* package as the slope of a linear model fitted with robust regression using repeated medians^99^. The relationship between the median hatching dates of sandeel and *C*. *helgolandicus* and these environmental variables were explored with linear models. The dependence of the mismatch measure, the overlap index and the recruitment index on the environmental variables and the abundance of age 1 sandeel was investigated with Generalized Linear Models (GLM) and GAM, using the *mgcv* package, to accommodate the conditional non-normal distribution of these indices and the non-linear nature of these relationships. All analyses were performed in the statistical software R 3.5.2^100^.

### Projected climate

The relationships between copepod and sandeel phenology and environmental variables were subsequently used to predict the effect of future climate on trophic mismatch between the two considered species. Two climate change scenarios were considered: a medium and a high GHG scenario. For the medium GHG scenario (Scenario A1B; IPCC, 2007)^101^, temperature projections near the seabed from a multi-level ocean model for UK waters (UKCP09: Land and marine past climate and future scenario projections data for the UK; http://catalogue.ceda.ac.uk/uuid/46f53c4e24f4428cba1c42a608844c82), centred on year 2080 and between latitudes 56.8 and 57°N and longitudes 1.8 and 2°W were used. For the high GHG model, predictions from the Scottish shelf model^102^, an unstructured grid 3D ocean model, with very high GHG emissions (RCP8.5^103^; were used. The predictions were for water temperature near the seabed in 2050 and at latitudes between 56.961 and 56.963° N and longitudes between 2.110 and 2.113°W.

## Supplementary information

Supplementary Material

## Data Availability

The datasets are available from the corresponding author on reasonable request.
