# Supplementary material for: Understanding temperature effects on recruitment in the context of trophic mismatch
Source: Sci Rep. 2019 Oct 23;9:15179. doi: 10.1038/s41598-019-51296-5 (PMC6811544; doi:10.1038/s41598-019-51296-5)
Supplement: Supplementary file 1 — Supplementary Material [file 41598_2019_51296_MOESM1_ESM.docx]

**Supplementary Information**

**Understanding temperature effects on recruitment in the context of trophic mismatch**

T. Régnier*, F. M. Gibb and P. J. Wright

*Marine Scotland Science, 375 Victoria Road, Aberdeen, AB11 9DB, Scotland, U.K.*

*Author to whom correspondence should be addressed. Tel: +44 (0)131 244 3084; email: [T.Regnier@marlab.ac.uk](mailto:T.Regnier@marlab.ac.uk)

**Supplementary Data tables and figures**

**Supplementary Table S1.** Median dates of *Calanus helgolandicus* copepodite V (CV) abundance observed from the Stonehaven time series and predicted from the distribution of adult stages (CVI) with the equations provided by Cook et al 2007 (CV duration = 0.1778 x 1/(8.106 .10^-5^(Temperature + 6.01)^2.05^). Difference in days between the observed and predicted dates is indicated as well as the average sampling intervals (in days).

| **Year** | **Observed CV Peak (Day of the Year)** | **Predicted CV Peak (Day of the Year)** | **Difference (days)** | **Average sampling interval (days)** |
| --- | --- | --- | --- | --- |
| **2000** | 131 | 126 | 5 | 7.1 |
| **2001** | 114 | 108 | 6 | 7.7 |
| **2002** | 119 | 117 | 2 | 8.4 |
| **2003** | 113 | 108 | 5 | 7.8 |
| **2004** | 104 | 107 | 3 | 8.0 |
| **2005** | 111 | 105 | 6 | 7.9 |
| **2006** | 114 | 111 | 3 | 8.3 |
| **2007** | 113 | 104 | 9 | 7.8 |
| **2008** | 120 | 110 | 10 | 7.6 |
| **2009** | 118 | 122 | 4 | 7.6 |
| **2011** | 136 | 128 | 8 | 8.9 |
| **2012** | 107 | 96 | 11 | 9.1 |
| **2013** | 137 | 126 | 11 | 8.4 |
| **2014** | 127 | 118 | 9 | 8.5 |
| **2015** | 128 | 124 | 4 | 8.0 |
| **2016** | 105 | 109 | 4 | 7.4 |
|  |  |  |  |  |


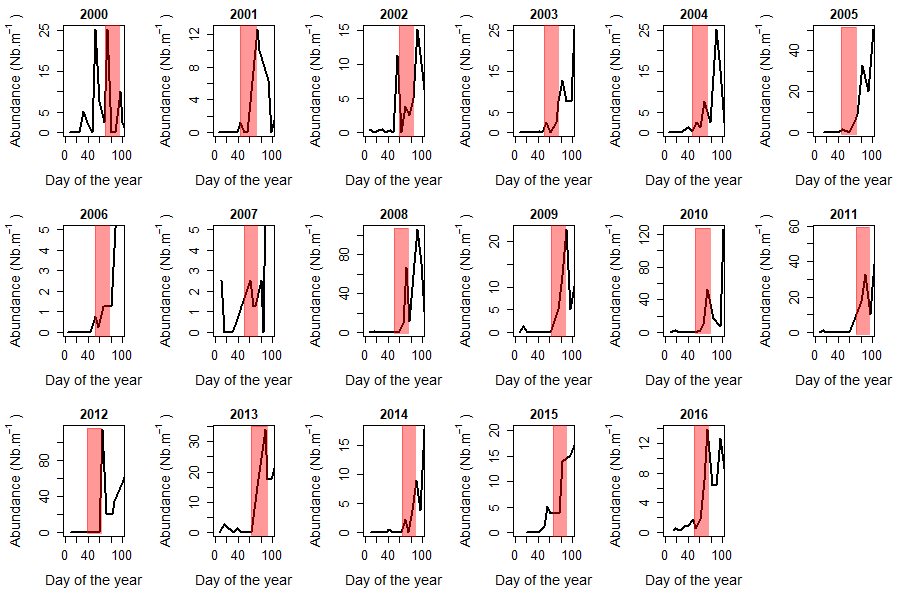


**Supplementary Figure S1.** Presence of large copepod naupliar stages. While the bongo nets fitted with a 200 micron mesh are not selective of naupliar stages, large nauplii (likely calanoid copepods of stages NI to NVI) can be retained in the sample. A presence or increase of nauplii during the back-calculated naupliar period for *Calanus helgolandicus* (red boxes) is in agreement with the model predictions.


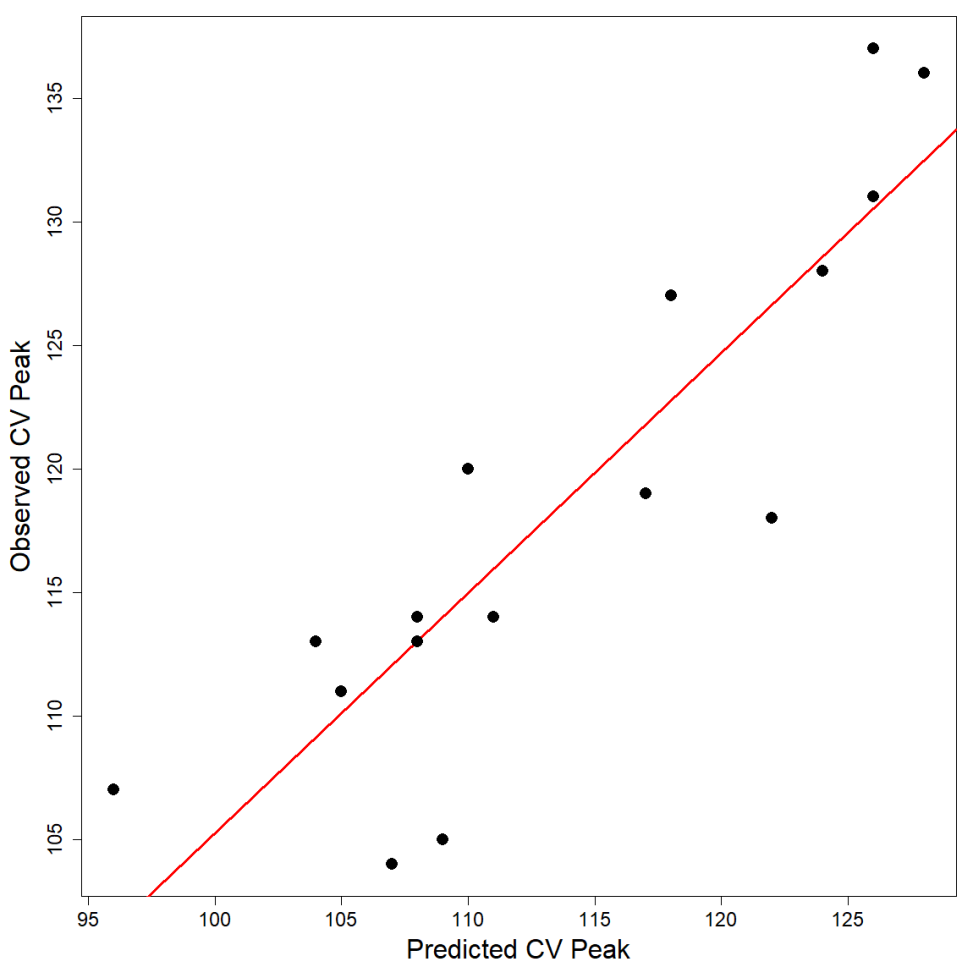


**Supplementary Figure S2. Agreement between observed and back-calculated phenology.** Relationship between observed date of peak copepodite V abundance and predicted dates calculated from copepodite VI abundances. The linear regression fir is indicated in red (F_1,14_ = 47, p <0.0001, r^2^= 0.77).
